# A Complete World Literature Review of Quality of Life (QOL) in Patients with Kidney Stone Disease (KSD)

**DOI:** 10.1007/s11934-016-0647-6

**Published:** 2016-10-22

**Authors:** Francesca New, Bhaskar K. Somani

**Affiliations:** Department of Urology, University Hospital Southampton NHS Trust, Southampton, SO16 6YD UK

**Keywords:** Quality of life, QOL, Kidney stone disease, KSD

## Abstract

**Purpose of Review:**

The purpose of this study was to review the current evidence for quality of life (QOL) in patients with kidney stone disease (KSD).

**Recent Findings:**

A review of literature from inception to May 2016 for all prospective English language articles on QOL in patients with KSD was done. QOL studies post urological procedures or ureteric stents were excluded. Nine studies (1570 patients) were included of which most (*n* = 6) used the SF-36 QOL tool. Overall, seven of the nine studies demonstrated a lower QOL in patients with KSD. Bodily pain and general health were significantly lower in patients with KSD compared to their control groups.

**Summary:**

Patients with KSD have an overall lower QOL with most impact on bodily pain and general health domains. Compared to the scale of patients suffering from KSD, more work needs to be done in measuring QOL both in terms of ‘Stone specific’ QOL measuring tools and the quality/number of studies in this field.

## Introduction

Kidney stone disease (KSD) is a common problem, affecting approximately 10–15 % of people in Europe and North America [1•]. In the USA, the lifetime prevalence for men is 12 %, and for women, it is 6 % [2]. Stone formers are 50 % more likely to have a further stone in the following 5 years [3]. Although some patients are asymptomatic with their KSD, many will have pain, urinary tract infection (UTI) or haematuria and may require multiple hospital admissions or multiple surgical procedures for this. This may also affect their renal function with an impact on their quality of life (QOL).

There are numerous ways to treat renal tract calculi, depending on their size, location, volume, anatomical factors and patient comorbidities. Historically, it was open surgical techniques; shock wave lithotripsy (SWL) was introduced in 1980, followed by percutaneous nephrolithotomy (PCNL) and subsequently endourological techniques with the popularisation of ureteroscopy (URS). After any one of these procedures, especially ureteroscopy, a ureteric stent may need to be placed. The presence of KSD, interventions for it and/or ureteric stents can all influence the QOL to varying degrees [4–10].

Patients with KSD can have increased levels of bodily pain, depression, loss of days at work and increased anxiety and financial distress, leading to overall lower QOL scores [11–15, 16••]. How KSD and its treatments affect QOL may affect patient or surgeon decisions regarding the management of their KSD [15]. The impact of KSD on patients’ QOL is becoming increasingly important to consider, as the focus of treatment has shifted not just only from considering morbidity and mortality but also considering the impact on their QOL [17–22, 23•].

Quality of life is a subjective experience and hence makes the effective measurement difficult. It is important to consider patients’ QOL, as it can help us understand how the disease affects their day to day living, and the personal burden of illness. This is not always related to the severity of their disease, by laboratory values or imaging, but by how the disease and possibly its treatment are perceived by the patient [18]. There are many psychosocial factors that need to be taken into consideration as well as symptom-related aspects of QOL. Examples of these are financial difficulties, stresses from job, family and associated pain [5]. There are a multitude of designed and validated tools used to measure this [5–8]. It is important for patients to assess their own QOL, not for health professionals to try and assume what it might be. Measuring QOL is important as one of the aims of any treatment is for the patient to feel and function normally. Using the information gathered from QOL studies, patients can be better informed on their treatment options and how they may fair after different treatments. Over the last 30 years, improving patients QOL has become an increasingly important part of treatment, and therefore, many tools have been produced to measure this [5–8]. However, there are currently no validated KSD-specific QOL tools available [15].

We conducted a systematic review of literature to look at the tools used for measuring QOL and the aspects of patients QOL most affected by KSD.

## Materials and Methods

### Evidence Acquisition

#### Criteria for Studies to Be Included in This Review

##### Inclusion Criteria

- Prospective studies written in the English language from inception to May 2016
- Studies reporting on QOL in patients with KSD

##### Exclusion criteria

- QOL studies of patients with ureteric stents
- QOL studies immediately after any urological procedure

Our aim was to look at the impact of KSD on patients’ QOL, which domains were affected, and to see which QOL tools were commonly used in urolithiasis patients.

### Search Strategy

The systematic review was performed according to the Cochrane reviews guidelines and the Preferred Reporting Items for Systematic Reviews and Meta-Analyses (PRISMA) guidelines [9]. We searched Pubmed, MEDLINE, EMBASE, Scopus, CINAHL, Cochrane library, Clinicaltrials.gov, Google Scholar and individual urological journals from inception to May 2016, and all English language articles were included in the original search. The search terms included: ‘Quality of life’, ‘kidney stone disease’, ‘urolithiasis’, ‘calculi’, ‘stones’ and ‘nephrolithiasis’. Boolean operators (AND, OR) were used with the above search terms to refine the search. Studies reporting on QOL in patients with KSD were included but studies on QOL in patients with ureteric stents or immediately after any surgical intervention were excluded. Data was extracted for the type of study, country of origin, review period, patient demographics, QOL tools used, domains measured and their effect on QOL.

## Results

### Literature Search and Included Studies

**Fig. 1:**
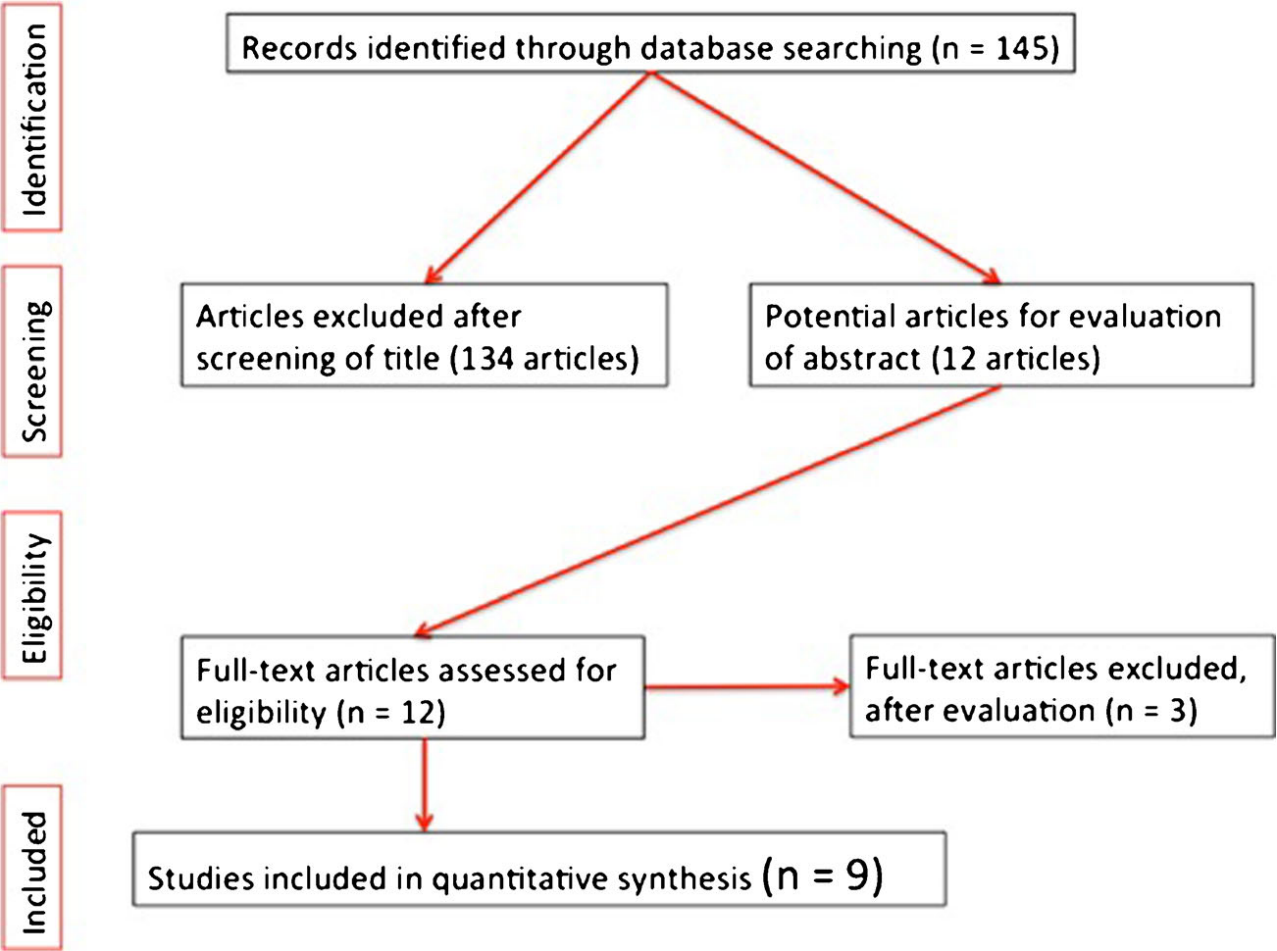
Inclusion criteria for final review of patients

After an initial search of 145 articles, 9 (1570 patients) met the inclusion criteria for the final review (Fig. 1). These were published from 2007 onwards, with six studies being from the USA. A full breakdown can be seen in Table 1.

**Table 1:** All studies reporting on KSD (included in our review)

Author	Year published	Journal	Review period	M:F	Mean age (years)	Patient number	QOL tool used
Chester J. Donnally III [11]	2011	Urology Research	2007–2009	1:1	51	152	SF-36
Kristina L. Penniston [16••]	2013	The Journal of Urology	2012	1:6	51	248	Winsicon stone QOL
Margaret S. Pearle and Yair Lotan [12]	2008	The Journal of Urology	2007	7:3	51	155	SF-36
Kristina L. Penniston and Stephen Y. Nakada [13]	2007	The Journal of Urology	1995–2006	1:1	51	189	SF-36
Jordan Angell, Michael Bryant [15]	2011	Journal of Urology	2005–2010	3:2	53	115	Emory stone questionnaire + CES-D
Denise H.M.P. Diniz^a^ Sérgio Luís Blay [14]	2007	Nephron Clinical Practice	2001–2004	1:2	44	194	SF-36
Bryant B, Angell J [18]	2012	The Journal of Urology	2005–2010	1:1	53	115	SF-36
Penniston KL [22]	2016	Journal of Endourology	2012	1:1	53	107	Winsicon stone QOL
Moderstikizi, F [23•]	2014	Urolithiasis	2014	1:1	47	295	SF36
			Total:	1:1	50	1570	

### Patient Characteristics and QOL tools used

In total, there were 1570 patients, with a mean age of 50 years (range 18–88 years). There was an even male to female distribution of 1:1. Most studies used the SF-36 QOL tool [11–14, 18, 23•] while two studies developed, and used a tool named the ‘Winsicon stone QOL tool’ [5, 15]. The final study used the Emory stone questionnaire (an aid to collect patient demographics, information about stones and procedures) and the CES-D depression questionnaire [16••, 24]. Eight of the studies were prospective in nature, all being level 2a/b in their evidence quality.

### Primary Outcomes

#### QOL Questionnaires Used

The main QOL tool used was the SF-36 questionnaire [11–14, 18, 23•]. It consists of 36 questions, which asses eight QOL domains; physical function (PF), role physical (RP), bodily pain (BP), general health (GH), Virility (V), social factors (SF), role-emotional (RE) and mental health (MH). It asks how these factors affected their life in the month preceding the questionnaire [5]. The three other studies used the Winsicon stone QOL tool [5, 16••] and the Centre for Epidemiologic Studies Depression Scale (CES-D) [15, 24]. The Winsicon stone QOL tool has 28 questions, which covered similar QOL aspects but also specifically asked about urinary frequency, dysuria and nocturia. The CES-D depression questionnaire is a 20-question survey used to illicit if patients have depressive symptoms.

#### Domains of QOL Measured

Six of the nine studies [11–14, 18, 23•] used the SF-36 questionnaire, a generic QOL tool, which divides patients QOL into eight domains. Five of these studies [12–14, 18, 23•] compared different QOL domains of patients with KSD to a case control group, or to the average QOL of the matched population. Four of these studies [13, 14, 18, 23•] reported the QOL scores for each domain (Table 2). Of these four, all demonstrated a lower QOL in patients with KSD, with Denise et al. demonstrating a statistically significant difference in all eight domains [14]. Byrant et al. showed a lower QOL in six of the eight domains (Physical Health, Bodily pain, General Health, Virility, Sexual Function) [18], while Kristina et al. showed lower QOL in general health and bodily pain [13]. Modersitzki et al. demonstrated a statistical significance in all eight domains within 1 month of a stone episode, with scores rising (QOL improving) over time from this episode [23•]. Bensalah et al. demonstrated significantly lower scores in five domains, including role physical, bodily pain, general health, social function and physical function [12].

**Table 2:** Studies using SF-36 matched with a case control

QOL domain (SF-36)	Diniz Sérgi, 2007 [14]		Kristina, 2007 [13]		Bryant M, 2012 [18]		Modersitziki F, 2014 [23•]	
	Stone patients (*p* value)	Case control	Stone patients (*p* value)	Case control	Stone patients (*p* value)	Case control	Stone patients (*p* value)	USA mean
Physical function	70(<0.05)	95	84(>0.05)	84	75(<0.001)	84	34(<0.001)	50
Role-physical	25(<0.05)	100	82(>0.05)	81	68(<0.001)	81	38(<0.001)	50
Bodily pain	41(<0.05)	84	69(<0.05)	75	67(=0.003)	75	44(<0.001)	50
Gen health status	52(<0.05)	82	65(<0.05)	72	60(=0.001)	72	32(<0.001)	50
Virility	45(<0.05)	80	59(>0.05)	61	53(<0.001)	61	34(<0.001)	50
Social function	63(<0.05)	100	85(>0.05)	83	78 (=0.01)	83	39(<0.001)	50
Role-emotional	33(<0.05)	100	86(>0.05)	81	78	81	34(<0.001)	50
Mental Health	54(<0.05)	84	75(>0.05)	75	74	75	33(<0.001)	50

The other three studies used alternative QOL tools. Angell et al. demonstrated clinically significant depression in 30.4 % of their patients with urolithiasis, where clinical depression was characterised as a CES-D score of 16 or more [16••]. The last two studies developed and used a specific QOL tool for patients with urinary tract stones. It contained 28 questions, looking at areas including irritability, fatigue, social impact, virility, urinary frequency and urgency, general health, physical pain and difficulty sleeping [16••]. Kristina et al. demonstrated that patients with active stones scored lower for the sum total of the questionnaire, than those who where asymptomatic [16••]. In the asymptomatic stone group, those with stones still scored lower in urinary frequency, urgency, general anxiety or nervousness about the future (*p* < 0.027) [5].

### Association Between Stone Episode and Time to Questionnaire Completion

Three out of the six studies documented average time from previous stone episode to questionnaire completion [12, 15, 16••]. The average time from these studies was 13 months (range 1–37 months). One study showed stability of SF-36 in KSD patients over a median follow-up of 18 months; however, a small cohort (*n* = 18) who had an acute stone episode within a month of completing their first questionnaire showed no significant differences in scores compared to other patients (*n* = 75) [11].

Byrant et al. demonstrated a significantly lower QOL for bodily pain and physical health domains in patients who had stone episode <1 month from completing the questionnaire [18]. A study on cysteine stone patients suggested that QOL gets better over a period of time and the timing of SF-36 needs to be accounted for when interpreting the domain scores and treatment, especially in patients with previous stone episodes [23•].

### Association Between Previous Stone-Related Procedure and QOL

Of the nine studies, two did not document any previous surgery for KSD [14, 15]. Seven studies documented previous surgical procedures for KSD, with an average of 64 % (43–80 %) of patients having prior stone surgery [5, 11–15, 23•]. Most suggest improvement of QOL over time especially in patients who suffered a recent or previous stone episode. Bensalah et al. analysed 155 patients from their clinic and found that the number of previous surgical interventions and body mass index had most affect on QOL especially their physical and mental components [12]. Similarly, another study using the SF-36 questionnaires on 115 patients suggested that the number of surgeries and surgical complications, time to stone episodes and the number of emergency room visits correlated most with the SF-36 physical and mental domains [18].

## Discussion

### Findings of Our Study

Overall, seven of the nine studies demonstrated a lower QOL in patients with KSD. Bodily pain and general health was significantly lower in patients with KSD compared to their control groups. There seems to be a correlation between stone episodes and QOL, and this seems to improve with the passage of time. Similarly, previous surgical intervention seems to have a negative impact on their QOL, as compared to the control group.

### Importance of Measuring QOL in KSD Patients

Patients with KSD tend to have a lower QOL even in the absence of stone episodes or interventions. It might reflect their previous experience of stone disease or an apprehension of the need for further treatment. Measurement of QOL is important to understand the impact of psychosocial and physical aspects of the disease. It can aid us in advising which management option may be more suitable for the individual. Only a longer-term follow-up over a few years would help us determine the time taken for the QOL domains to get back to baseline. QOL measurements also help us to evaluate and see ways in which we can improve our surgical choices or technique to improve patients’ QOL [21].

### Comparison and Outcomes of Different QOL Studies

There are a multitude of generic QOL tools; selecting a measure can be difficult, as there are so many to choose from [4]. Examples of generic available measures are Short Form 36 (SF36) [5], Hospital and Anxiety Depression Scale (HADS) [6] and Profile of Mood States (POMS) [7]. There is also a QOL tool for patients with a ureteric stent in situ, the ureteric stent specific questionnaire (USSQ) [8]. The four different tools that where used in the literature in this review all have their own advantages and disadvantages that are summarised in Table 3. None of the tools used so far are perfect for assessing the QOL of patients with KSD. Large numbers of patients suffer with KSD [1•, 2, 3], and it has a huge impact on a person’s QOL [11–15, 16••, 18]. A disease-specific QOL tool that is universally used would be useful to measure and compare QOL in these patients.

**Table 3:** Advantages and disadvantages of current questionnaire used

Questionnaire	Advantages	Disadvantages
SF-36 [5]	• Covers wide range on QOL domains • Widely used	• No KSD specific questions
Winsicon QOL in stones [16••]	• Stone specific • Treatment specific	• Not validated • Large questionnaire • Not broken into domains • Difficult to analyse
Emory stone questionnaire [15]	• Demographic specific • Stone specific	• Not a QOL measurement
CES-D [24]	• Specific for depression	• No QOL domains

The most common QOL tool used in our literature review for patients with KSD was the SF-36. As the SF-36 is a generic questionnaire, it does not target symptoms specific to stone formers and may not be sensitive enough to measure their QOL accurately [11]. However. when analysing the studies using the SF-36 questionnaire, we found a statistically significant difference in the bodily pain and general health sub domains (Table 2).

There are many disease-specific QOL tools [25], although we could find none specifically designed for patients with KSD that had been widely validated. One study [15] aimed to fill this gap and produce a tool (the Winsicon Stone Quality of life questionnaire), specifically for patients with KSD. They also looked at asymptomatic stone formers in a paper published in 2016, and they found that even if the person was not aware of having KSD, but did have stones, they still had a lower QOL in specific domains, particularly urinary frequency, urgency, anxiety or nervousness (*p* = <0.027) [22]. In the limitations of these two studies, the authors identified that further research into this area needs to include understanding the role of comorbidities and social economic status in patients with both symptomatic and asymptomatic stones, as well as identifying the need for multi-institutional testing of the WiSQoL questionnaire to validate it.

### Limitations of the Study

The studies using the same QOL questionnaires did not assess the data in a similar fashion, or compared the patients QOL to the same ‘norm’. None of the studies made it clear if the patient was having an active stone at the time of questionnaire administration, or in the month prior (the SF-36 only measure QOL in the 31 days prior to completing the questionnaire). It is well recognised that recent procedures and ureteric stents lower patients’ QOL and to avoid bias, we did not include these studies in our review [17].

Some of the other limitations are the lack of stone characteristics in the data provided, such as size, position and composition of stones. There were no randomised controlled trials and all studies were of level 2a/b evidence.

A number of confounding factors associated with KSD can also affect QOL of these patients. For example, obesity has been shown to lower QOL [19] and is also known to be associated with stone formers. One of the studies demonstrated that QOL in stone formers was worse in women and in patients with high BMIs [13]. Chronic diseases such as gout, diabetes, inflammatory bowel disease and bowel procedures are all associated with stone formation, but may themselves lower patients QOL [20, 26, 27]. Other patient-related confounding factors that may impact on measurement of QOL includes difficulty completing the questionnaire, procedural and judgement issues. Even with these limitations, seven [5, 12–15, 16••, 23•] out of the nine studies demonstrated lower QOL in patients with KSD.

### Areas of Future Research

Areas of future research could include evaluating the WisQOL questionnaire over a larger and multi-institutional patient cohort. It would also be of benefit to look not just at health-related QOL, including the physical, mental and emotional burden of KSD to health, but also the financial impact, including the loss of earnings to the individual as well as the financial cost on the health service. Donnally et al. in their longitudinal evaluation of QOL using SF-36 found no significant changes in domains suggesting that a validated disease-specific questionnaire might be better in these patients [11].

An important aspect of KSD is the affect to patients’ family and wider concept of management of other associated medical conditions either related to or contributing to KSD. Any QOL study is perhaps incomplete without addressing some of these factors. It is perhaps time that research and resource is allocated to generating patient-reported QOL outcome measures specific to KSD.

## Conclusion

KSD affects QoL in most patients with most impact on bodily pain and general health domains. Compared to the scale of patients suffering from KSD, more work needs to be done in measuring QOL both in terms of ‘Stone specific’ QOL measuring tools and the quality/number of studies in this field.
